# Defects in Mitochondrial Fission Protein Dynamin-Related Protein 1 Are Linked to Apoptotic Resistance and Autophagy in a Lung Cancer Model

**DOI:** 10.1371/journal.pone.0045319

**Published:** 2012-09-20

**Authors:** Kelly Jean Thomas, Marty R. Jacobson

**Affiliations:** 1 Biological Sciences Department, Colorado Mesa University, Grand Junction, Colorado, United States of America; 2 Saccomanno Research Institute, St. Mary’s Hospital and Regional Medical Center, Grand Junction, Colorado, United States of America; University of Medicine and Dentistry of New Jersey, United States of America

## Abstract

Evasion of apoptosis is implicated in almost all aspects of cancer progression, as well as treatment resistance. In this study, resistance to apoptosis was identified in tumorigenic lung epithelial (A549) cells as a consequence of defects in mitochondrial and autophagic function. Mitochondrial function is determined in part by mitochondrial morphology, a process regulated by mitochondrial dynamics whereby the joining of two mitochondria, fusion, inhibits apoptosis while fission, the division of a mitochondrion, initiates apoptosis. Mitochondrial morphology of A549 cells displayed an elongated phenotype–mimicking cells deficient in mitochondrial fission protein, Dynamin-related protein 1 (Drp1). A549 cells had impaired Drp1 mitochondrial recruitment and decreased Drp1-dependent fission. Cytochrome c release and caspase-3 and PARP cleavage were impaired both basally and with apoptotic stimuli in A549 cells. Increased mitochondrial mass was observed in A549 cells, suggesting defects in mitophagy (mitochondrial selective autophagy). A549 cells had decreased LC3-II lipidation and lysosomal inhibition suggesting defects in autophagy occur upstream of lysosomal degradation. Immunostaining indicated mitochondrial localized LC3 punctae in A549 cells increased after mitochondrial uncoupling or with a combination of mitochondrial depolarization and ectopic Drp1 expression. Increased inhibition of apoptosis in A549 cells is correlated with impeded mitochondrial fission and mitophagy. We suggest mitochondrial fission defects contribute to apoptotic resistance in A549 cells.

## Introduction

Cancer is a major public health problem around the world. Current strategies in cancer therapy (chemo- and radiotherapy) rely on killing tumor cells by mechanisms largely mediated by the activation of apoptosis. Apoptosis is a conserved cellular process that controls normal development and tissue homeostasis by eliminating damaged cells. Inhibition of apoptosis contributes to the tumorigenic conversion of normal cells by extending their viability, favoring the accumulation of transforming mutations [1]. Resistance to apoptosis is linked to increased invasive and metastatic potential in cancer cells [2].

A classic cancer hallmark is apoptotic resistance [3]. How tumor cells evade apoptotic cell death is currently unknown, but increasing tumor sensitivity to apoptosis is a therapeutic goal. The absence of spontaneous apoptosis and treatment-induced apoptosis in non-small cell lung cancer (NSCLC) suggests that deficiencies in the apoptotic process may be responsible for their resistance to anti-cancer therapy [4]. Gene mutations and altered expression of apoptosis regulators are detected in lung cancer. Differences in sensitivity to therapeutics that induce apoptosis may be related to the expression of apoptosis regulators in lung cancer. Anti-apoptotic modulating therapy is being extensively investigated [3].

Intrinsic apoptosis is characterized by permeabilization of mitochondria, release of cytochrome c, and activation of the caspase cascade [5]. Mitochondrial control of apoptosis occurs upstream of caspase activation and is mediated by the Bcl-2 family of proteins [5]. Bcl-2 proteins also influence mitochondrial dynamics, a process that balances mitochondrial fission and fusion events to regulate the shape, structure and function of the mitochondrion [5]. Mitochondria are dynamic organelles whereby their shape corresponds to the metabolic status [6], the health of the cell [7] and balance between fusion and fission is required for homeostasis. Normally, mitochondrial fission mediator Drp1 fragments mitochondria [5]. Drp1 is a large GTPase that controls membrane tubulation and fission in mammalian cells [5]. Cells undergoing mitochondrial fission will have shorter mitochondrial length when compared to cells that are undergoing mitochondrial fusion [8]. Although these events are transient, cells deficient in a mitochondrial dynamics protein or under stimuli will maintain their state-dependent mitochondrial morphology [6]. Excessive mitochondrial fission (fragmentation) is essential for intrinsic apoptosis–it is necessary for cytochrome c release and subsequent caspase activation [5].

Inhibition of Drp1-dependent mitochondrial fission impairs and partially inhibits intrinsic apoptosis [7]. Concomitant with mitochondrial membrane permeabilization during apoptosis, Drp1 forms oligomers and is recruited to the outer mitochondrial membrane to mediate fission in a GTP-dependent manner [5]. Fission events mediate apoptosis by regulating the release of pro-apoptotic factors to the cytosol. Inhibition of Drp1 prevents mitochondrial membrane potential collapse and cytochrome c release, and promotes elongated mitochondrial morphological phenotypes [5]. Inhibition of mitochondrial fission prohibits cytochrome c translocation and delays cell death, thus providing a link between mitochondrial dynamics and the induction of apoptosis.

Mitochondrial dynamics not only impact intrinsic apoptosis, but also redirect autophagic degradation. Extensive cross-talk exists between mitophagy and apoptosis [9]. Inhibition of fission mediators such as Dynamin-related protein 1 (Drp1), which impairs intrinsic apoptosis [10], has been shown to decrease mitophagy [11]. Downregulation of autophagy during tumor progression has been noted in many studies [12]. Increased tumorigenicity has been shown to decrease protein degradation in lung epithelial cells [13]. The negative regulator of autophagy, mTOR (mammalian target of rapamycin), is frequently activated [14] and mTOR inhibitors have been shown to limit tumor proliferation in NSCLC models [15].

Resistance to apoptosis is linked to mitochondria and proteins involved in mitochondrial dynamics. However, it remains unknown if tumor cells gain resistance to apoptosis by altering mitochondrial dynamics. In this study, we characterized mitochondrial dynamics and the downstream process of apoptosis in lung cancer cells. Differences in mitochondrial morphology and function were observed in A549 cells. We provide evidence in lung cancer cells suggesting an imbalance in mitochondrial dynamics exists, whereby defects in Drp1-dependent mitochondrial fission inhibit the downstream process of autophagy and contribute to apoptotic resistance.

## Materials and Methods

### Cell Culture and Plasmids

Cell lines purchased from ATCC (Table I) were cultured as described previously [16]. Live cell imaging was performed in phenol red free (PRF) OptiMEM (Invitrogen). Plasmids [16] for mitochondrial YFP (mito-YFP, Clontech), Drp1-YFP [17], Drp1-myc [18], Drp1 K38A-myc [18], and Drp1 RNAi [19] were gifts from Dr. Richard Youle (NINDS, Bethesda, MD) and were transfected into cells using Lipofectamine 2000 (Invitrogen) as directed by the manufacturer. Plated cells (10^4^ cells/well) were seeded and cultured in complete OptiMEM for 24 hours at 37°C, prior to treatment and fluorescent intensity reading. Cells treatment included: Incubation with 1 µM staurosporine (STS, Sigma) and/or 50 µM zVAD-FMK (Sigma) for 3 hours in PRF OptiMEM without serum in apoptotic assays. Cells were starved using EBSS (Invitrogen) and/or treated with 10 nM Bafilomycin A1 (Sigma) for 24 hours in autophagy assays.

**Table 1:** Lung epithelial cell lines used.

Designation	ATCC #	Tissue	Disease	Tumor type	Tumorigenic (Ref)
NL20	CRL-2503	bronchis	normal	N/A	No [49]
NL20TA	CRL-2504	bronchis	normal	N/A	Yes, weakly [49]
Calu1	HTB-54	lung	squamous cell carcinoma	pleural metastasis	Yes [50]
A549	CCL-185	alveolar	adenocarcinoma	1° explant lung culture	Yes [51]

### Analysis of Mitochondrial Morphology and Connectivity

Mitochondrial morphology was analyzed as previously described [16] using live cell imaging with an inverted confocal microscope (CARV spinning disk, DMI 6000B, Leica) and MetaMorph software. Mitochondrial length analysis was performed on images using ImageJ after processing to find edges. The average mitochondrial length was determined after using the freehand line selection tool to measure length (micrometers) of individual mitochondria obtained in a randomly selected field of 100×100 pixels and 10–15 cells per cell line were examined. Associated metadata were displayed and the image scale (Distance in Pixels: 1; Known Distance: Value from Metadata; Pixel Aspect Ratio: 1; Unit of Length: micrometer; Global: checked) was set for image analysis consistency. Transient cell transfection and Drp1 RNAi knockdown experiments were performed as described [16]. Mitochondrial imaging with 0.5 µg transfected mito-YFP or mito-DsRED, mitochondrial length estimates and mitochondrial FRAP assays have been described [16], [20].

### Cellular Imaging and Fluorimetry

Mitochondrial mass was estimated using a fluorescent plate reader after staining cells with 200 nM Mitotracker Green for 30 minutes at 37°C, and washing once with PRF-OptiMEM [21]. Fluorescence intensity measurements reported are background subtracted (495/515 nm).

Mitochondrial membrane potential was estimated in a 96-well plate using a fluorescent plate reader (Tecan). Cells (10^4^ per well) were loaded with 10 nM TMRE for 30 minutes at 37°C in normal growth media. The TMRE loading medium was removed and cultures were placed in phenol red free, serum free media and incubated for an additional 15 minutes to allow for the TMRE fluorescent signal to reach steady state. Fluorescence intensity was then captured 1/min for 5 minutes to establish a baseline. After 5 minutes, 6 µM oligomycin (Cell Signaling) was added to induce hyperpolarization of Δψ_m_. In the presence of oligomycin, 10 µM CCCP was added at 18 minutes to the cells to cause depolarization of the ΔΨ_m_. TMRE expression is plotted as a relative ratio ΔF/F_0_ showing mean and SEM, where F indicates fluorescence intensity and F_0_ indicates baseline values before stimulation. All cell lines analyzed displayed oligomycin-induced hyperpolarization of mitochondrial membrane potential. Fluorescence intensities are background subtracted (554/576 nm).

Mitophagy was measured using confocal microscopy to detect colocalization of mito-YFP transfected positive cells with immunostaining against mouse monoclonal anti-LC3B (Novus Biologicals) with AlexaFluor® 594 secondary anti-mouse IgG antibody (Invitrogen). Cells (5×10^4^ per well) were transfected in suspension with 0.5 µg mito-YFP using Lipofectamine 2000 (Invitrogen) and seeded on LabTekII borosilicate glass chambers (Nunc) for 24 h. After fixation with 4% paraformaldehyde, cells were immunostained with rabbit polyclonal anti-LC3B antibody (Novus Biologicals) and visualized with AlexaFluor® 594 goat anti-rabbit IgG (590/617 nm). The number of distinct mitochondria localized LC3-positive punctae per cell was counted. Autophagosome formation was noted by LC3 punctae.

All fluorescence measurements have relative fluorescence units (RFU).

### ELISA, Phosphoprotein Purification and Western Blotting

General methods for western blotting and subcellular fractionation have been described [20]. Antibodies used: Sigma (β-actin, GAPDH), Cell Signaling (caspase-3, HSP60, HTRA2, PARP), BD Translabs (Drp1, p53, TIM23), Calbiochem (VDAC), Novus (LC3-II), Abcam (vimentin, fibronectin) and MitoSciences (Mitobiogenesis Assay, complex IV subunit I, frataxin). The antibody against Drp1 pS637 was a generous gift from Dr. Craig Blackstone (NINDS, Bethesda, MD). Phosphoprotein purification was performed as previously described [16].

### Statistics

Tests for analysis are indicated in the figure legend and significance is denoted as follows: ns, not significant *P*>0.05; * *P*<0.05; ** *P*<0.01; ***P<0.001.

## Results

### Mitochondrial Elongation in A549 Cells

**Figure 1:**
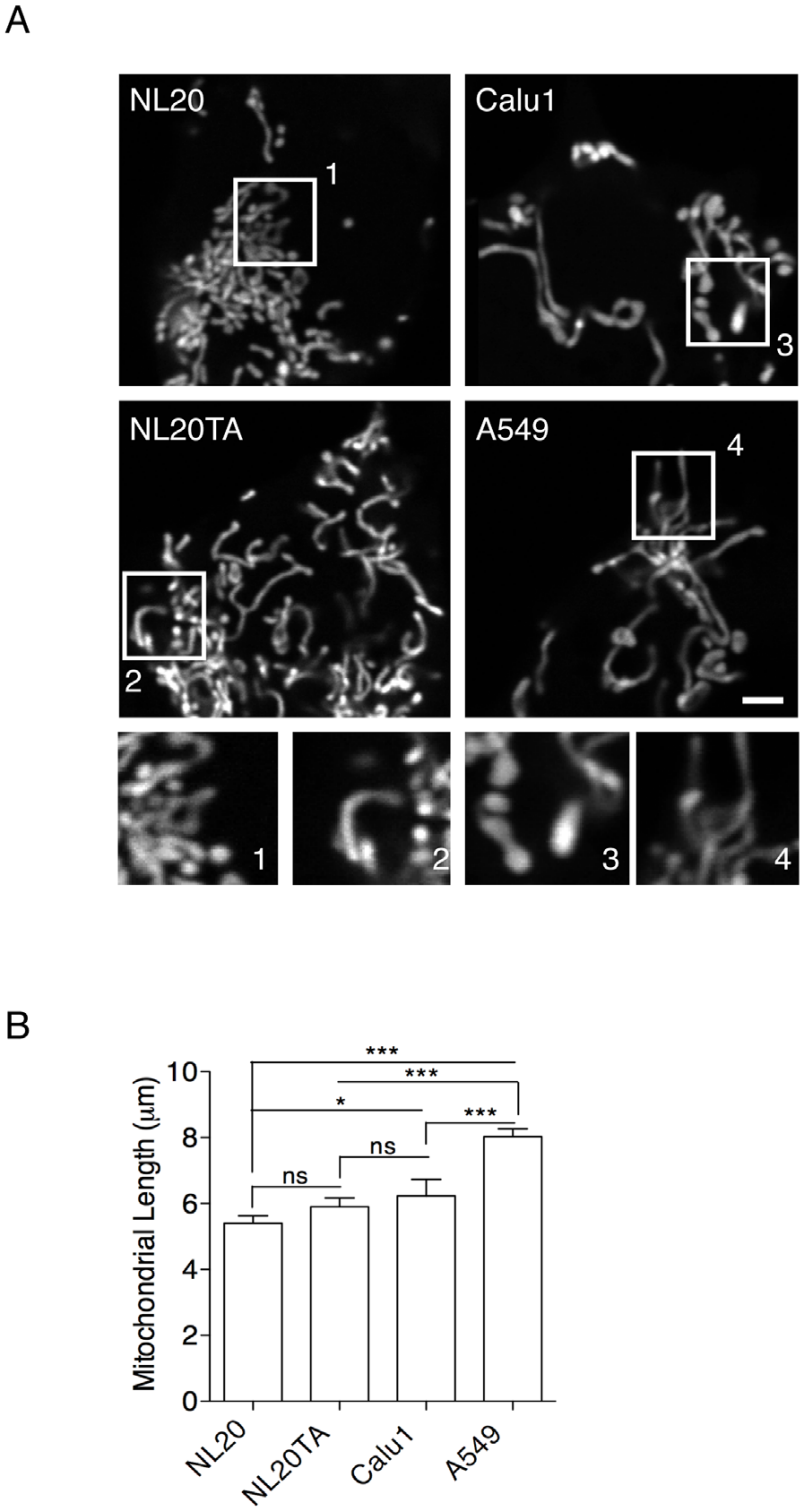
Mitochondrial length of lung epithelial cells. (A) Representative mito-YFP expression in cells. Numbered boxes (1–4) are magnifications (10×) of the corresponding numbered boxed regions in the original images of NL20, NL20TA, Calu1 and A549. Scale bar is 2 µm. (B) Mean mitochondrial length (y-axis; ± SEM) measurements (n = 35–50) from three independent experiments for NL20, NL20TA, Calu1, and A549 cells. 1-way ANOVA analysis with Tukey post-tests (*P*<0.0001).

Mitochondrial dysfunction has been observed in many types of cancer cell lines [22]. An important determinant of mitochondrial function is mitochondrial morphology. Mitochondrial morphology was examined in each of our normal and lung cancer cell lines (Table I) using a mito-YFP to fluorescently label the mitochondrial matrix of cells (Figure 1A). Mitochondrial length was also determined for each cell line (Figure 1B). Distinct differences in the morphology, including the length of mitochondria, were observed between lung epithelial cell lines that vary in tumorigenic potential (Table I) and relative tumorigenic potential of each cell line was validated after performing cell migration experiments (Figure S1) [23]. The non- and weakly tumorigenic NL20 and NL20TA cells had a heterogeneous phenotype but the tumorigenic Calu1 and A549 cells displayed predominantly the elongated form of mitochondria. A trend was noted whereby the non-tumorigenic cells (NL20) had the shortest mean mitochondrial length (mean±SEM; 5.2±0.4 µm) and the longest mean mitochondrial length was observed in A549 cells (mean±SEM; 8.0±0.8 µm) (Figure 1).

### Increased Mitochondrial Mass in A549 Cells

**Figure 2:**
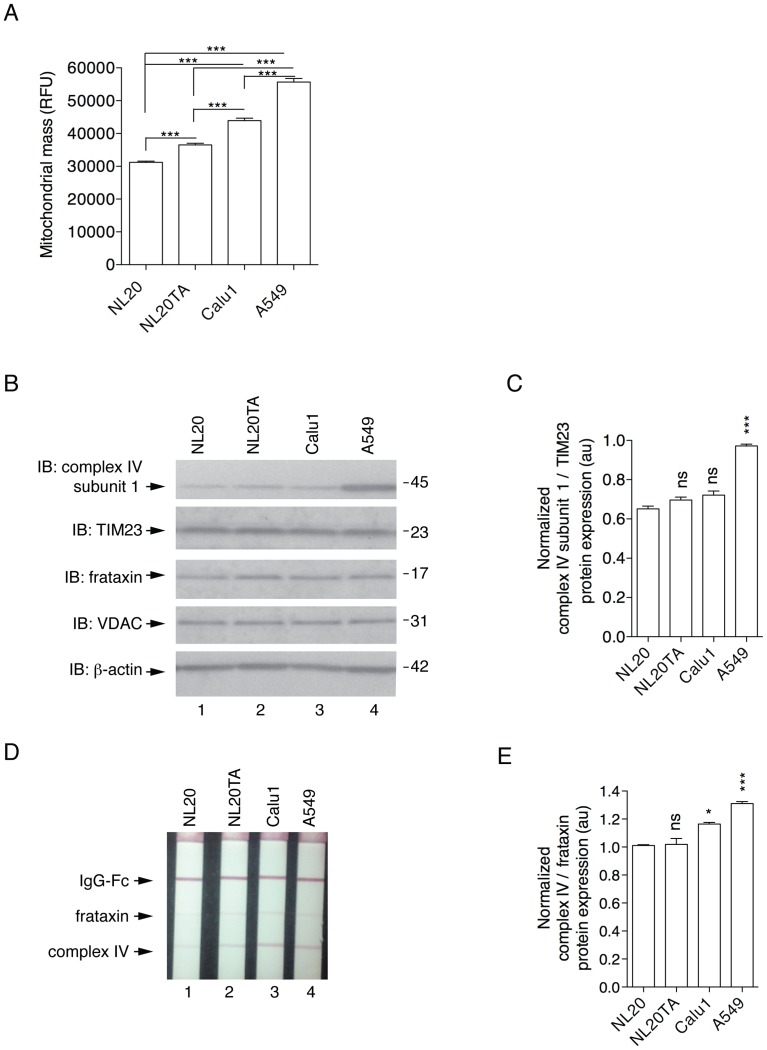
Mitochondrial mass of lung epithelial cells. (A) Mitotracker green fluorescence relative fluorescence units (y-axis, RFU) represents mitochondrial mass (mean and SEM); measurements (n = 8 wells) from three independent experiments. 1-way ANOVA analysis with Tukey post-tests (*P*<0.0001). (B) Representative immunoblots demonstrating expression of endogenous proteins complex IV subunit I, TIM23, frataxin, and VDAC in NL20 (lane 1), NL20TA (lane 2), Calu1 (lane 3), and A549 (lane 4) cells. β-actin is shown for loading. Markers in kilodaltons (kDa). (C) Immunoblots from two independent experiments were quantified to show relative complex IV subunit I protein expression normalized to TIM23. Mean and SEM shown. 1-way ANOVA analysis with Tukey post-tests compared to NL20. (D) Representative mitochondrial biogenesis dipstick ELISA assay showing frataxin and complex IV expression. (E) Three independent mitochondrial biogenesis ELISA assays were quantified to show relative mitochondrial-encoded complex IV protein expression normalized to the nuclear-encoded frataxin protein. Mean and SEM shown. 1-way ANOVA analysis with Tukey post-tests compared to NL20.

Mitochondrial mass was estimated in live cells using Mitotracker Green FM, which accumulates in mitochondria regardless of mitochondrial membrane potential [24]. After normalization for cell volume, it was observed that A549 cells had increased mitochondrial mass compared to the other lung epithelial cell lines (Figure 2A). To further validate this relationship, the expression of a mitochondrial encoded protein, mitochondrial complex IV subunit I (the terminal enzyme of the respiratory chain) was compared to nuclear encoded mitochondrial-associated proteins, TIM23 (translocase of the inner mitochondrial membrane 23) and frataxin by two independent methods, immunoblot and ELISA (Figure 2B,D; respectively). A549 cells had increased levels of complex IV subunit I protein when compared to NL20 cells (Figure 2B–E). The selective accumulation of mitochondria in A549 cells was not mimicked by other organelles upon analysis of protein expression (ER, calnexin; golgi, syntaxin 6; cytosol, GAPDH) by western blot (data not shown). Additionally, mitochondrial biogenesis markers TFAM (mitochondrial transcription factor A) and PGC1α (peroxisome proliferator-activated receptor gamma coactivator-1 alpha) were examined to further investigate mitochondrial mass in the cell lines. Examination of these markers at the gene expression level does not suggest that increases in mitochondrial mass observed in A549 cells are attributed to increased mitochondrial biogenesis. Gene expression fold change differences were not significant in both gene evaluations (*TFAM*, *P*<0.142; *PGC1α*, *P*<0.119) (data not shown). These data show A549 cells have increased mitochondrial mass.

### Resistance to CCCP-induced Mitochondrial Depolarization in A549 Cells

Mitochondrial uncoupling by CCCP (carbonyl cyanide m-chloro phenyl hydrazone) disrupts mitochondrial membrane potential (Δψ_m_) and mitochondrial pH gradient (ΔpH_m_) [20]. TMRE expression is plotted as a relative ratio, ΔF/F_0_, where F indicates fluorescence intensity and F_0_ indicates baseline values before stimulation. All cell lines analyzed displayed oligomycin-induced hyperpolarization of mitochondrial membrane potential. In the presence of oligomycin, 10 µM CCCP was then added to the cells to cause depolarization of the Δψ_m_.

**Figure 3:**
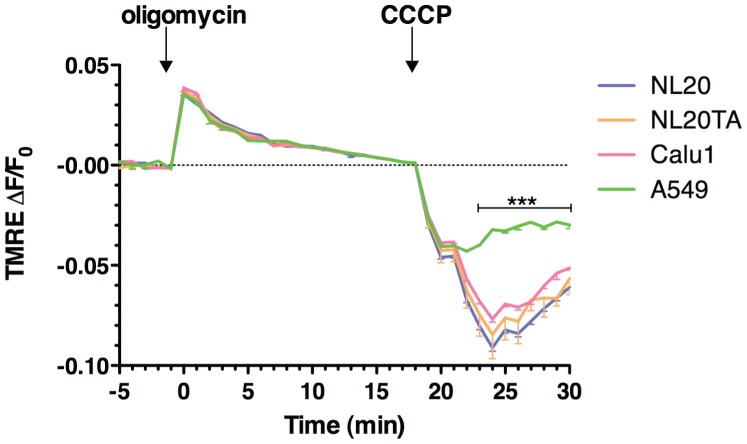
Resistance to mitochondrial uncoupling. TMRE staining from two independent experiments (n = 8). TMRE expression is plotted as a relative ratio ΔF/F_0_ showing mean and SEM, where F indicates fluorescence intensity and F_0_ indicates baseline values before stimulation following background subtraction**.** Cell lines NL20 (blue), NL20 (orange), Calu1 (pink1) and A549 (green) were treated with oligomycin (6 µM; 0 minutes) and CCCP (10 µM; 18 minutes) to induce hyperpolarization and depolarization, respectively. 2-way ANOVA analysis with Bonferroni post-tests.

Our data suggest that A549 cell lines do not depolarize to the level of the other cell lines following CCCP treatment. Significant differences (P<0.001; 2-way ANOVA) in ΔF/F_0_ are observed from 23–30 minutes (>4 min after CCCP addition) between A549 and the other cell lines. This suggests that highly tumorigenic A549 cells display a resistance to mitochondrial membrane depolarization (Figure 3).

As tumor cell lines have previously been shown to be influenced by multidrug resistance transporters (MDR), cells were pre-treated with 50 µM verapamil to block MDR activity during TMRE loading to determine if MDR proteins in the plasma membrane had an effect on TMRE influx. Following oligomycin/CCCP treatment of verapamil treated cells, MDR did not influence the intracellular accumulation of TMRE when compared to non-verapamil treated cells (data not shown); as this fluorescent rhodamine substrate does not accumulate in drug resistant cells. In this study, A549 cells had less change in Δψ_m_ as measured by TMRE fluorescence following CCCP treatment and MDR activity does not influence this activity. These data are consistent with other reports that suggest cancer cells demonstrate resistance to mitochondrial membrane depolarization [25].

### Decreased Drp1-dependent Fission in A549 Cells

Mitochondrial depolarization and fission are a prerequisite for intrinsic apoptosis [5]. With increased mitochondrial length, we hypothesized that A549 cells would have decreased mitochondrial fission, which would decrease both apoptotic initiation and the downstream catabolic process of autophagy.

**Figure 4:**
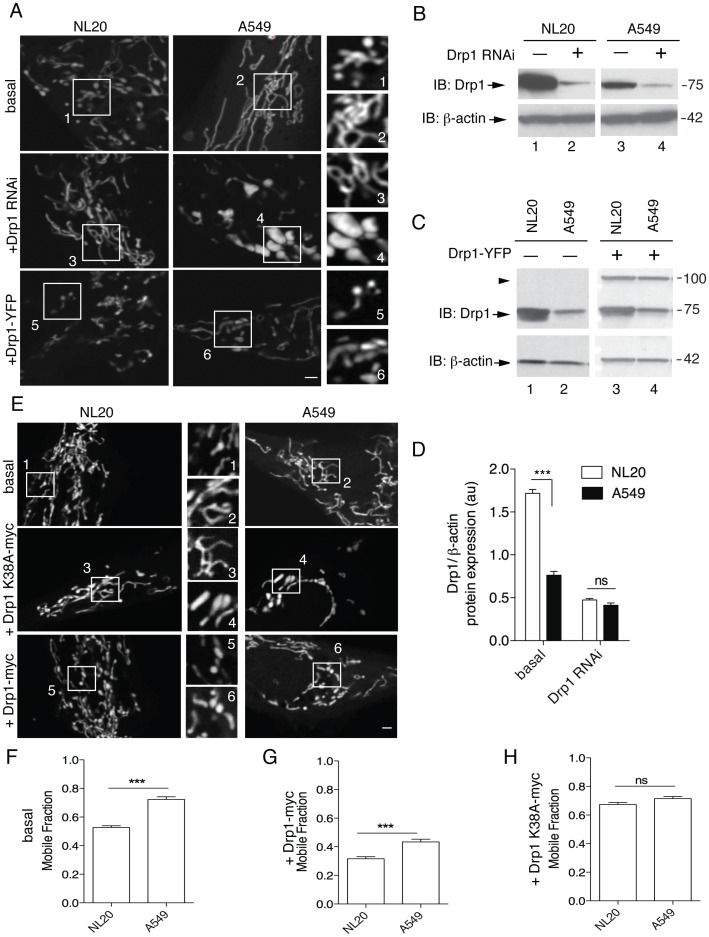
Decreased Drp1 protein expression and Drp1-dependent mitochondrial fission. (A) Mitochondrial morphology of NL20 (left) and A549 (right) cells following mito-DsRED transfection. Representative images shown: basal (upper) and following Drp1 RNAi (middle) or Drp1-YFP transfection (lower). The scale bar indicates 2 µm. Numbered boxes (1–6) are magnifications (10×) of the corresponding numbered boxed regions in the original images. (B) Immunoblot of endogenous Drp1 expression before (lanes 1,3) and after (lanes 2,4) Drp1 RNAi transfection in NL20 and A549 cells. (C) Immunoblot of endogenous (lanes 1,2; arrow) and ectopic (lanes 3,4; arrowhead) Drp1 expression before and after Drp1-YFP transfection in NL20 and A549 cells. (B,C) β-actin reprobe to show loading; markers in kDa. (D) Relative Drp1 protein expression normalized to β-actin before and after Drp1 RNAi transfection in NL20 (white bar) and A549 (black bar) cells (two independent experiments). 2-way ANOVA analysis with Bonferroni post-tests. (E) Representative images from FRAP analysis of NL20 (left) and A549 (right) cells transfected with mito-YFP. Cells imaged under basal (top), Drp1 K38A-myc downregulation of Drp1 (middle) and Drp1-myc overexpression (bottom) conditions. Numbered boxes (1–6) are magnifications (10×) of the corresponding numbered boxed regions in the original images. Scale bar is 1 µm. (F–H) Mobile fraction of mito-YFP values occurring within a single subcellular region of interest (n = 60 cells). Mean and SEM shown from two independent experiments. 1-way ANOVA analysis with Tukey post-tests. Additional FRAP statistics are shown in Figure S2.

Drp1 and its mitochondrial fusion antagonists Opa1 (Optic atrophy 1), Bax/Bak and Mfn1/2 (Mitofusin 1/2), in part, regulate the shape, structure and function of the mitochondria by mitochondrial dynamics [8]. Examination of mitochondrial morphology (Figure 1A; Figure 4A, basal) shows mainly elongated mitochondria in A549 cells (Figure 4A2: mitochondrial length, mean±SEM; 8.3±0.8 µm). This phenotype suggests defects in fission or an upregulation of fusion. Steady state Drp1 protein levels were analyzed by immunoblot (Figure 4B,C). Quantitative analysis shows that basal Drp1 protein expression in A549 cells was at most 44% that observed in NL20 cells (Figure 4D).

To further manipulate mitochondrial morphology, Drp1 was knocked down using RNAi [16], [19](Figure 4A,B +Drp1 RNAi), which is expected to elongate mitochondria. After Drp1 RNAi mediated knock down in NL20 and A549 cells, there was a robust decrease in Drp1 protein levels (Figure 4B,D). Mitochondrial length increased, as expected, in NL20 cells after Drp1 RNAi treatment (Figure 4A: mean±SEM; basal (A1), 4.9±0.5 µm; Drp1 RNAi (A3), 7.8±0.6 µm; 1-way ANOVA, Tukey post-test, *P*<0.001). The mitochondrial phenotype after Drp1 RNAi treatment in A549 cells was swollen and bulbous (Figure 4A4); a morphology caused by hyperfusion of the mitochondria due to a lack of balance that is normally provided by fission [26]. To determine if reduced Drp1 protein expression contributes to the elongated mitochondrial phenotype normally observed in A549 cells, cell lines were transfected with Drp1-YFP plasmid to overexpress Drp1-YFP fusion protein, which is functionally capable of inducing fission [16]. As shown in Figure 4A (+Drp1-YFP), Drp1 overexpression rescued the mitochondrial phenotype in A549 cells, decreasing mitochondrial lengths to those observed in basal NL20 cells (Figure 4A: mean±SEM; +Drp1-YFP (A6), 5.1±0.7 µm; 1-way ANOVA, Tukey post-test, *P*>0.05). The mitochondrial morphology of A549 cells following Drp1 overexpression similarly resembled that of basal NL20 cells (Figure 4A1). Mitochondrial length in NL20 cells (Figure 4A: mean±SEM; basal (A1), 4.9±0.5 µm) also decreased after Drp1 overexpression (Figure 4A: mean±SEM; +Drp1-YFP (A5), 2.1±0.3 µm; 1-way ANOVA, Tukey post-test, *P*<0.001).

These data suggest that a deficiency in Drp1 protein levels contributes to decreased mitochondrial fission events in A549 cells. FRAP (fluorescence recovery after photobleaching) was used to quantify mitochondrial connectivity, which infers mitochondrial fission events [16], in living NL20 and A549 cells (Figure 4E–H). FRAP curves were summarized by calculating the mobile fraction of mitochondrial-directed yellow fluorescent protein (mito-YFP), which estimates mitochondrial connectivity [16]. FRAP experiments confirm that A549 cells have decreased mitochondrial fission (Figure 4F–H). Mobile fraction values show that mitochondria in NL20 cells have significantly lower functional connectivity (more fission) when compared to A549 cells basally (Figure 4F). Downregulation of Drp1 by overexpression of a dominant negative version of Drp1 (Drp1 K38A) equilibrated the mitochondrial connectivity between the two cell lines (Figure 4H). Following Drp1 overexpression (+Drp1-myc), mitochondrial fission is enhanced in A549 cells such that mobile fraction values are more comparable to basal NL20 cells (Figure S2). These FRAP results mimic the mitochondrial morphological observations which show decreased Drp1-dependent fission in A549 cells when compared to NL20 cells and that downregulated fission in A549 cells can be rescued by overexpression of Drp1.

### Drp1 Localization in A549 Cells

**Figure 5:**
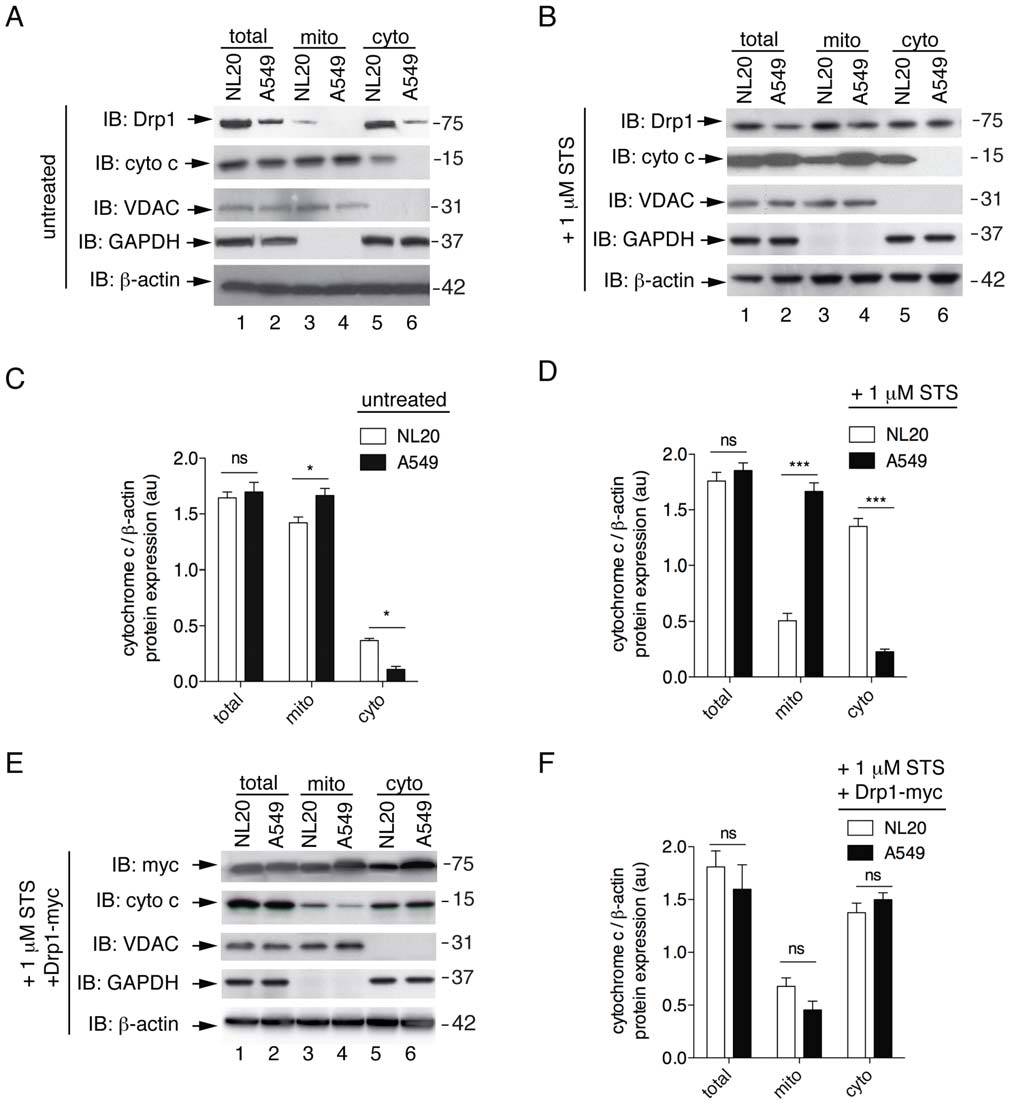
Impaired release of cytochrome c following apoptotic stimulus with STS. (A,B, E) Representative immunoblots of three independent experiments of (A) untreated, (B) 1 µM STS treatment for 3 h to induce apoptosis, and (E) 1 µM STS treatment for 3 h after Drp1-myc transfection in NL20 and A549 cells. Endogenous Drp1 and cytochrome c protein expression were evaluated in total (lanes 1,2), mitochondrial (lanes 3,4) and cytosolic (lanes 5,6) fractions. Mitochondrial (VDAC), cytosol (GAPDH) and β-actin (total) are included as fractionation and loading controls. Myc expression after Drp1-myc transfection is shown in (E). Markers in kDa. (C, D, F) Cytochrome c protein expression normalized to β-actin in NL20 (white bars) and A549 (black bars) cell fractions under untreated conditions (C), 1 µM STS treatment for 3 h (D) or 1 µM STS treatment for 3 h with ectopic expression of Drp1-myc (F). Mean and SEM shown from two independent experiments. 2-way ANOVA analysis with Bonferroni post-tests. Mitochondrial morphology under these conditions is shown in Figure S3C and S5.

To further examine decreased Drp1-dependent fission in A549 cells, we looked at Drp1 localization following subcellular fractionation via immunoblot. Mitochondrial and cytosolic fractions were examined for endogenous Drp1 protein levels (Figure 5A,B). Drp1 protein was not observed in the mitochondrial fraction of A549 cells suggesting that Drp1 is not actively recruited to the mitochondria for fission. In contrast, NL20 cells showed recruitment of Drp1 to the mitochondrial fraction, which infers mitochondrial fission is occurring in NL20 cells as suggested by the mitochondrial morphological analyses (Figure 1A, 4A) and confirmed by FRAP data (Figure 4F–H). In mammalian cells, Drp1 is recruited to the outer mitochondrial membrane following apoptotic induction [5]. To examine this translocation, Drp1 localization was visualized by immunoblot following subcellular fraction after staurosporine (STS) treatment. STS is a protein kinase inhibitor known to induce apoptosis [27]. Upon apoptotic induction by STS, Drp1 protein recruitment from the cytosolic to the mitochondrial fraction was observed in both NL20 and A549 cells (Figure 5B). From this observation, we inferred that A549 cells have decreased Drp1 mitochondrial recruitment that can be enhanced by apoptotic stimulation. This suggests that Drp1 can be recruited to the mitochondria in A549 cells but the process of mitochondrial fission is impeded in this cell line.

### Impaired Cytochrome C Release in A549 Cells

Inhibiting mitochondrial fission delays the release of cytochrome c into the cytoplasm [28]; therefore, we examined cytochrome c translocation in A549 cells (Figure 5, Figure S3). Mitochondrial and cytosolic fractions were examined for cytochrome c release in NL20, NL20TA, Calu1 and A549 cells with and without CCCP-induced uncoupling (Figure S3A,B). Specifically, A549, the most tumorigenic cell line in this panel (Figure S1), displayed impairment in cytochrome c release following CCCP treatment (Figure S3B,C). STS-treated A549 cells showed a similar lack of cytochrome c release when compared to NL20 cells (Figure 5B, Figure S3C). Immunoblots were quantified to show cytochrome c protein levels in fractionation experiments without and with STS-treatment (Figure 5C,D). Under basal and STS conditions, A549 cells had increased cytochrome c protein levels in the mitochondrial fraction when compared to NL20 cells. These data suggest A549 cells have decreased Drp1-dependent mitochondrial fission and the downstream process of cytochrome c release is impaired. To further support this idea, overexpression of DRP1 in A549 cells rescued STS-treatment induced release cytochrome c in this cell line (Figure 5E,F; Figure S3C) demonstrating that impaired cytochrome c release observed in A549 cells is due to a deficiency in Drp1. Expected mitochondrial morphological phenotypes were observed following STS-treatment and in combination with Drp1-myc overexpression (Figure S5), supporting the cytochrome c release immunoblot data in NL20 and A549 cells.

### Apoptotic Resistance in A549 Cells

**Figure 6:**
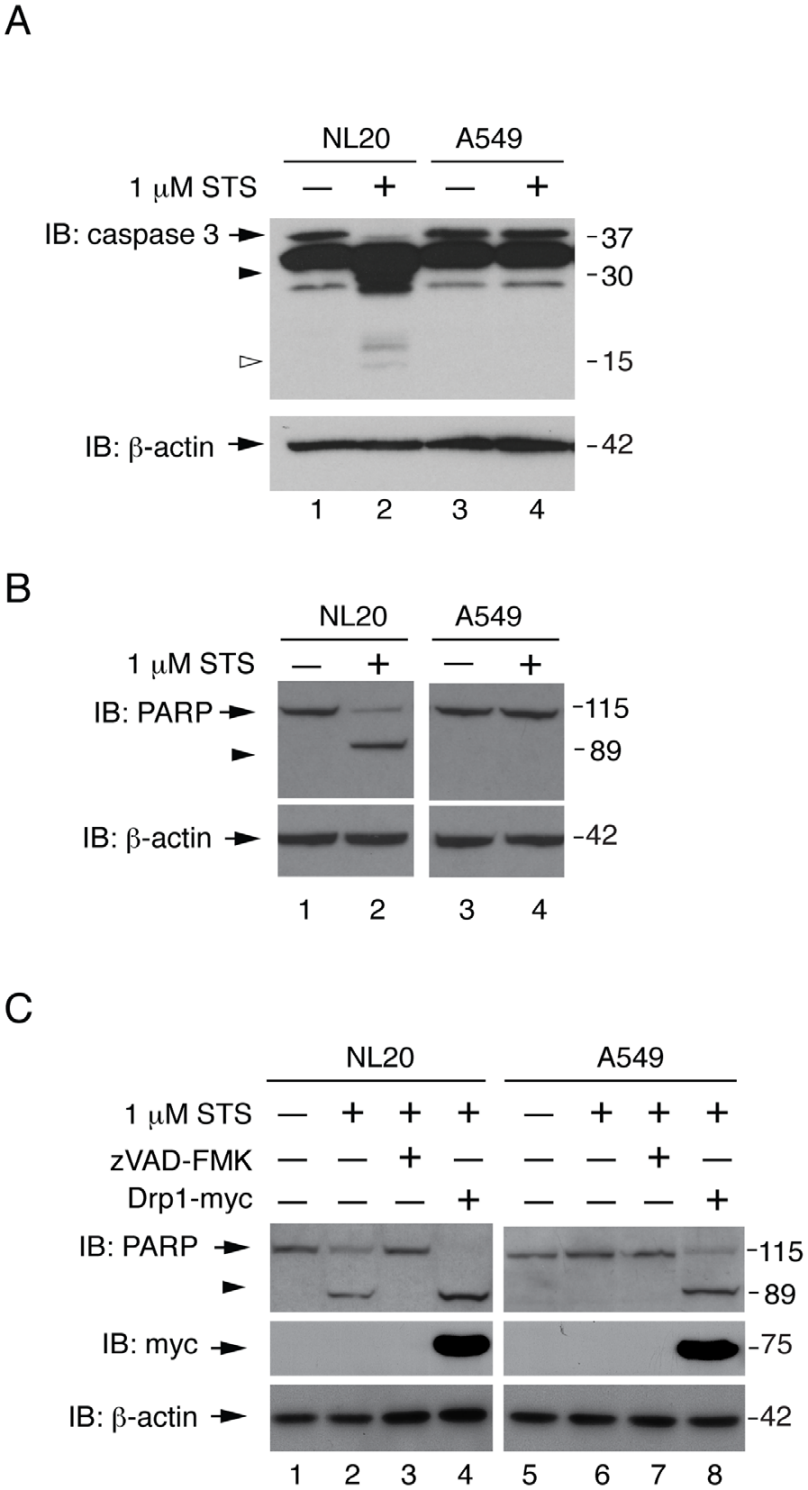
Resistance to STS-induced caspase-3 and PARP cleavage. (A, B) NL20 and A549 cells treated with (lanes 2,4) and without (lanes 1,3) 1 µM STS for 3 hours to induce apoptosis. β-actin reprobe to show loading. Immunoblots show (A) endogenous caspase-3 protein expression; full length (arrow) and cleaved products (open or closed arrowhead). (B) Endogenous PARP protein expression; full length (arrow) and cleaved product (arrowhead). (C) Endogenous PARP protein expression; full length (arrow) and cleaved product (arrowhead) in NL20 and A549 cells treated with 50 µM zVAD-FMK (lanes 3,7) for 4 h to inhibit caspase activity in non-transfected (lanes 1–3, 5–7) or Drp1-myc transfected (lanes 4,8) cells or co-treatment with 1 µM STS for 3 h (lanes 2–4,6–8). (A–C) Markers in kDa.

Since mitochondrial fission defects delay cytochrome c release [28] and caspase activation [5], we examined downstream pro-apoptotic events following cytochrome c release. Cleavage of caspase-3, a key mediator of mammalian apoptosis [29], was observed following STS- and doxorubicin-treatment (Figure 6A, Figure S4A,C) in NL20, NL20TA, and Calu1 cells. In contrast, caspase-3 cleavage was absent in apoptosis stimulated A549 cells (Figure 6A; Figure S4A). PARP cleavage, a downstream component of apoptosis that is caused in part by activation of caspase-3 [30], was also examined following apoptotic induction (Figure 6B, Figure S4B,D). PARP cleavage was observed in NL20 cells following apoptotic stimuli but was absent in A549 cells. Inhibition of STS-induced PARP cleavage in NL20 cells was observed upon concomitant treatment with zVAD-FMK (Figure 6C), a known caspase inhibitor, demonstrating that this is a caspase mediated process in NL20 cells. Complete PARP cleavage was observed in STS-treated NL20 cells overexpressing Drp1 protein (Figure 6C). STS-treatment induced PARP cleavage was restored in A549 cells overexpressing Drp1 (Figure 6C). These data suggest that A549 cells are resistant to apoptosis due to a downregulation of Drp1 protein expression.

### Impaired Mitophagy in A549 Cells

There is a relationship between mitochondrial fission and the induction of mitochondrial selective autophagy, also known as mitophagy [31]. During mitophagy, the pre-autophagosome engulfs mitochondria to form an autophagosome. Concurrently, the cytosolic protein microtubule-associated protein 1A/1B-light chain 3 (LC3-I, 18 kDa) is conjugated to phosphatidylethanolamine to form LC3-phosphatidylethanolamine conjugate (LC3-II, 16 kDa) [32]. LC3-II is then recruited to autophagosomal membranes prior to fusion with the lysosome, which then proteolytically degrades autophagosomal internal components including LC3-II [32]. As such, lysosomal turnover of the autophagosomal marker LC3-II reflects autophagic events, such as mitochondrial selective degradation by autophagy, and detecting LC3 by immunoblotting or immunofluorescence is the standard for monitoring autophagy [11], [33].

**Figure 7:**
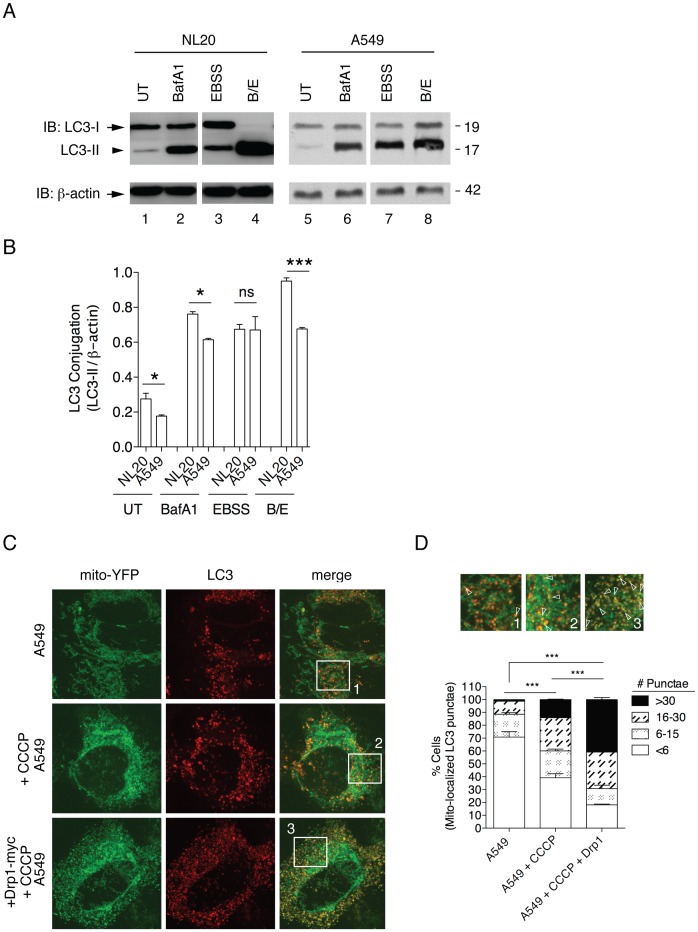
Impaired turnover of mitochondria prior to degradation by the lysosome. (A) Representative immunoblot of LC3-I (arrow) to LC3-II (arrowhead) conversion in total protein from untreated (UT; lanes 1,5), 10 nM 24 h Bafilomycin A1 treated (BafA1; lanes 2,6) 24 h serum starved (EBSS; lanes 3,7) or combined 24 h BafA1/EBSS treated (B/E; lanes 4,8) NL20 and A549 cells. β-actin reprobe to show loading. (B) LC3-II quantification (normalized to β-actin) from two independent experiments. Cells conditions were as in (A) to examine differences between (B) cell lines. Mean and SEM shown, 2-way ANOVA analysis with Bonferroni post-test. (C) Mito-YFP transfection (left) and LC3 immunostaining (middle) in A549 cells show mitophagy by colocalization of LC3 with the mitochondria (merged, right). A549 cells treated with 10 µM CCCP for 30 minutes (middle) or transfected with Drp1-myc for 24 h and CCCP treated (bottom). Representative images of mitophagy by colocalization of mito-YFP and LC3 punctae (yellow expression) in CCCP and CCCP/Drp1-myc conditioned A549 cells. Scale bar is 2 µm. (D) Quantification of mitophagy as in (C). Box insets show enlarged microscopy sections to illustrate a selection of mitophagy events occurring (white open arrowheads) which are labeled 1–3 corresponding to original images as shown in (E); A549 (1), A549+CCCP (2), and A549+CCCP/Drp1-myc (3). Percent cell counts (>200) from three independent experiments were based on colocalization of LC3 punctae staining with mito-YFP (values represent mean number of punctae counts per cell binned with SEM). 2-way ANOVA analysis with Bonferroni post-tests.

Previous studies have shown that defects in mitochondrial fission decrease mitochondrial degradation or mitophagy [11]. Autophagy was assessed by immunoblot and immunostaining (Figure 7). We monitored steady state LC3-I to LC3-II conjugation by immunoblotting for endogenous LC3 (Figure 7A) [20]. Less LC3-II conjugate was observed in untreated A549 cells when compared to NL20 cells (Figure 7B–D), which suggests basal autophagy is decreased in A549 cells relative to NL20 cells. Serum starvation, which induces autophagy, was used as a positive control. No significant difference in the amount of LC3-II following serum starvation was observed between NL20 and A549 cell lines following serum starvation (Figure 7B, EBSS), suggesting that A549 cells possess the functional capacity for autophagy. Cells were also treated with Bafilomycin A1 (BafA1), which prevents lysosomal degradation and results in the increased accumulation of LC3-II and autophagosomes [20]. NL20 cells showed efficient autophagic flux as demonstrated by the presence of more LC3-II banding than LC3-I following BafA1 treatment (Figure 7A). Because the level of LC3-II is not greater than LC3-1 in BafA1 treated A549 cells, A549 does not show efficient autophagic flux and we can infer that autophagy is impaired prior to degradation by the lysosome in this cell line (Figure 7A). To further support this data an LC3 turnover assay was performed (Figure 7A; lanes 4,8), which combines serum starvation and lysosomal inhibition to monitor flux [34]. A complete turnover of LC3-II was noted in NL20 cells, whereas A549 cells had similar LC3-II expression levels as observed with only Bafilomcyin treatment. These results further support autophagy being limited in A549 cells. To confirm this observation in another technique, autophagosomes were noted by the detection of LC3 punctae and mitophagy was inferred by the colocalization of LC3 punctae with mito-YFP expression using confocal microscopy (Figure 7C). A significant difference in the percentage of cells with <6 mitochondrial localized LC3 punctae was identified between basal and CCCP-treated A549 cells (Figure 7D). This observation shows that depolarization of the mitochondria after CCCP treatment does not significantly induce mitophagy in A549 cells. However, following Drp1 overexpression in A549 cells and subsequent treatment with CCCP, the percentage of cells undergoing mitophagy increased significantly (Figure 7D). These data suggest that A549 cells have impaired mitophagy.

## Discussion

In this study, A549 cells displayed elongated mitochondrial phenotypes basally and hyperfused mitochondria following downregulation of Drp1, consistent with phenotypes observed in cells that are deficient in mitochondrial fission [5], [35]. Additional similarities to cells lacking Drp1 were noted in A549 cells, such as resistance to mitochondrial depolarization and impaired cytochrome c release from the mitochondria [5]. Drp1 activity can be altered by mitochondrial recruitment, post-translational modification and its oligomerization state [5]. This study shows decreased expression and recruitment of Drp1 to mitochondrial fractions in A549 cells relative to non-tumorigenic NL20 cells, and decreased Drp1-dependent mitochondrial fission. Drp1 associates with mitochondrial fission sites upon oligomerization [5]. Because Drp1 was observed in mitochondrial fractions following apoptotic stimuli (Figure 5C,D), we presumed Drp1 oligomerization was intact in A549 cells. To determine if phosphorylation of Drp1 was involved in the observed inhibition of mitochondrial fission in A549 cells, we examined the phosphorylation status DRP1 in both A549 and NL20 cells (Figure S6). We noted a significant increase in the phosphorylation of Drp1 in A549 cells, which would inhibit GTPase activity and subsequent fission events [5]. This phosphorylation data supports the hypothesis that A549 cells have decreased mitochondrial fission.

Defects in mitochondrial dynamics have been implicated in numerous degenerative neurological disorders [36]. The disruption of an antagonistic response by Drp1 in mitochondrial dynamics could potentially impact many key cellular events, such as development, apoptosis, autophagy, synaptic plasticity and cell division [37]. Extensive cross-talk exists between autophagy and apoptosis [38]; in non-tumorigenic cells, the deletion of genes regulating autophagy is associated with decreased apoptosis [9]. Accordingly, alterations in apoptosis and autophagy were observed in tumorigenic A549 cells.

Efficient removal of damaged mitochondria by mitophagy requires fission of the mitochondrial network [11]. Turnover of damaged mitochondria stimulates mitochondrial biogenesis to replenish each cell with a stable pool of functional mitochondria. With respect to metabolic function, changes in mitochondrial mass can be indicative of mitochondrial proliferative potential or turnover [39]. In A549 cells, we observed an increased mitochondrial mass, which may indicate a lack of mitochondrial degradation by mitophagy [40]. We inferred defects in mitophagy by decreased LC3-II protein expression observed in A549 cells. Decreased mitophagy is influenced by Drp1 expression, as mitophagy is rescued in A549 cells following Drp1 overexpression.

Recent literature supports our findings whereby mitochondria elongate and are spared from autophagic degradation and sustain viability [41], purposing that regulated changes in mitochondrial morphology determine the fate of the cell during autophagy. As the role of autophagy in tumor progression and treatment is controversial [42], perhaps regulation of mitochondrial morphology is indeed the determinant factor in the cellular response to autophagy.

The data presented fit our hypotheses, which suggest that elongated mitochondrial lengths observed in A549 cells correlate with decreased mitochondrial fission events, and subsequent decreases in both apoptotic initiation and autophagy in this tumorigenic cell line. This work is novel in that we have identified defects in mitochondrial dynamics, namely in the mitochondrial fission mediator Drp1, which contribute to tumorigenic conversion in lung cancer cells. To summarize, tumorigenic A549 cells exhibit mitochondrial dynamic imbalances, which downregulate pro-apoptotic signaling in these tumorigenic lung epithelial cells allowing deregulated cells to survive. We also demonstrate decreased Drp1 protein expression and increased Drp1 protein phosphorylation in A549 cells, relative to the normal lung epithelial cell line NL20, which allows substandard mitochondria to accumulate causing defects in mitochondrial clearance, which potentially impacts further mitochondrial function and tumorigenesis.

Decreased Drp1 expression and activity observed in A549 cells could be due to transcriptional regulation. Drp1-dependent fission has been shown to be inhibited by miR-30 family members through suppression of its modifier, the *p53 tumor suppressor* gene [43]. It is noteworthy that A549 cells express the wild-type version of *p53* [44] and p53 protein expression in these cells is not statistically different from NL20 cells (data not shown). In addition to post-transcriptional gene silencing, other RNA polymerase modifiers may be regulating Drp1 gene transcription and subsequent activity.

Other possible hypotheses of Drp1 regulation include further post-translational modifications. As we have inferred, like others have demonstrated [45], [46], Drp1 mediated fission is negatively regulated by phosphorylation. Drp1 function has also been shown to be modulated in part by calcineurin-dependent dephosphorylation [16]. Tumorigenic A549 cells could potentially have decreased phosphatase enzymatic activity or constitutively active Drp1 phosphorylation, both of which would limit GTP-dependent mitochondrial fission. Moreover, sumoylation of Drp1 has been shown to positively regulate its activity and mitochondrial morphology [47], [48]. Downregulation of sumoylation players may also putatively inhibit mitochondrial fission in tumorigenic cells. Drp1 is also modified by ubiquitination [5], a process that targets the protein for destruction. This modification could potentially influence the turnover of Drp1, which might explain the significant decrease in protein expression observed in A549 cells when compared to controls.

Future studies in A549 cells will examine the influence of Drp1 modifiers including the cristae-remodeling pathway, controlled in part by Opa1, on the downstream processes of mitochondrial dynamics, autophagy and apoptosis in tumorigenic lung epithelial cells.

## Supporting Information

Figure S1 **Tumorigenic potential measured by cell migration assay.** Percent cell migration was assessed in NL20, NL20TA, Calu1 and A549 cells using a standard Boyden chamber assay. Mean and SEM shown from triplicate (n = 8 cell measurements) experiments. 1 way ANOVA analysis with Tukey post-tests compared to NL20 cells (*P*<0.0001).
(TIF)

Figure S2 **Additional FRAP analysis.** Mobile fraction of mito-YFP values in NL20 and A549 cells are displayed which estimate mitochondrial connectivity or the relative amount of mitochondrial fission that is occurring in a single region of interest within the cell under basal, Drp1 K38A-myc downregulation or Drp1-myc overexpression. Mean and SEM shown from duplicate (n = 60 cell measurement) experiments. 1-way ANOVA analysis with Tukey post-tests.
(TIF)

Figure S3 **Cytochrome c release following mitochondrial uncoupling.** (A,B) NL20 (lanes 1–3), NL20TA (lanes 4–6), Calu1 (lanes 7–9), and A549 (lanes 10–12) cells were harvested and subcellular fractionation was performed to examine mitochondrial and cytosolic fractions. Total (T: lanes 1,4,7,10), mitochondrial (M: lanes 2,5,8,11) and cytosolic (C: lanes 3,6,9,12) lysates were immunoblotted for endogenous cytochrome c. The mitochondrial (VDAC) and cytosol (GAPDH) markers and β-actin are shown as loading and fractionation controls. Markers are in kDa. (B) Cells were treated with 10 µM CCCP for 1 h to induce mitochondrial decoupling to examine cytochrome c release. (C) NL20 (left panels) and A549 (right panels) cells were transfected with mito-dsRED and immunostained for cytochrome c to show mitochondrial (red/orange) or cytoplasmic (green) localization. Colocalization of mitochondria and cytochrome c is indicated by yellow. Cells were untreated (top panels), treated with 1 µM STS for 3 h (second panels), treated with 10 µM CCCP for 1 h (third panels), co-transfected with Drp1-myc (fourth panels) or co-transfected with Drp1-myc and treated with 1 µM STS for 3 h (bottom panels). Scale bar is 2 µm.
(TIF)

Figure S4 **Apoptotic stimulus in epithelial cells.** (A,B) NL20 (lanes 1,3) and A549 (lanes 2,4) cells untreated (lanes 1,2) or treated for 24 h with 1 µg/ml doxorubicin (lanes 3,4). β -actin reprobe to show loading. (C,D) NL20TA (lanes 1,2) and Calu1 (lanes 3,4) cells untreated (lanes 1,3) or treated for 3 h with 1 µM STS (lanes 2,4). NL20TA (lanes 5,6) and Calu1 (lanes 7,8) cells untreated (lanes 5,7) or treated for 24 h with 1 µg/ml doxorubicin (lanes 6,8). β -actin reprobe to show loading. (A,C) Immunoblots show endogenous caspase 3 protein expression (full length, arrow; cleaved products, open or closed arrowhead). (B,D) Immunoblots show endogenous PARP protein expression (full length, arrow; cleaved products, arrowhead). (A–D) Markers in kDa.
(TIF)

Figure S5 **Mitochondrial morphology following STS exposure.** (A) Mitochondrial morphology of NL20 (left panels) and A549 (right panels) cells following mito-YFP transfection. Representative images shown: basal (upper panels), and following 3 h treatment with 1 µM STS (middle panels) or Drp1-myc co-transfection with 3 h 1 µM STS treatment (lower panels). The scale bar indicates 2 µm. Box insets (numbered 1–6) to the far right are magnified (10×) regions corresponding to the numbered boxes in the original images.
(TIF)

Figure S6 **Analysis of Drp1 phosphorylation.** (A) Cell lysates from NL20 and A549 cells were separated by phospho-enrichment (P; lanes 1,3) and compared to total lysates (T; lanes 2,4). With immunoblot, endogenous Drp1 was found in both phospho- and total fractions. Immunoblotting for Drp1 pSer637 was also included. Controls for purification include immunoblotting for non-phosphorylated HSP60 and phosphorylated HTRA2. Markers in kDa. (B) The ratio of phospho- to total Drp1 was quantified (B) from two independent experiments between the two cell lines. Mean and SEM shown. T-test analysis (*P* = 0.0071).
(TIF)
