# Supplementary figures and images for: Defects in Mitochondrial Fission Protein Dynamin-Related Protein 1 Are Linked to Apoptotic Resistance and Autophagy in a Lung Cancer Model
Source: PLoS One. 2012 Sep 20;7(9):e45319. doi: 10.1371/journal.pone.0045319 (PMC3447926; doi:10.1371/journal.pone.0045319)

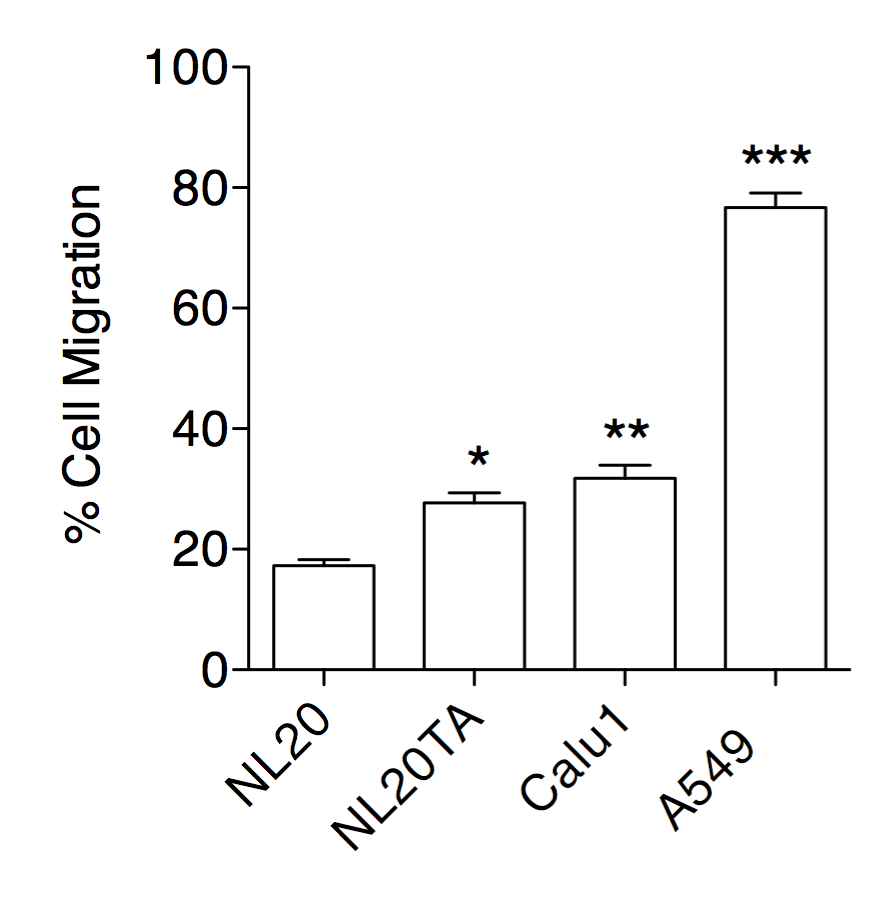

Supplement: Figure S1 — Tumorigenic potential measured by cell migration assay. Percent cell migration was assessed in NL20, NL20TA, Calu1 and A549 cells using a standard Boyden chamber assay. Mean and SEM shown from triplicate (n = 8 cell measurements) experiments. 1 way ANOVA analysis with Tukey post-tests compared to NL20 cells (P<0.0001). (TIF) [file pone.0045319.s001.tif]

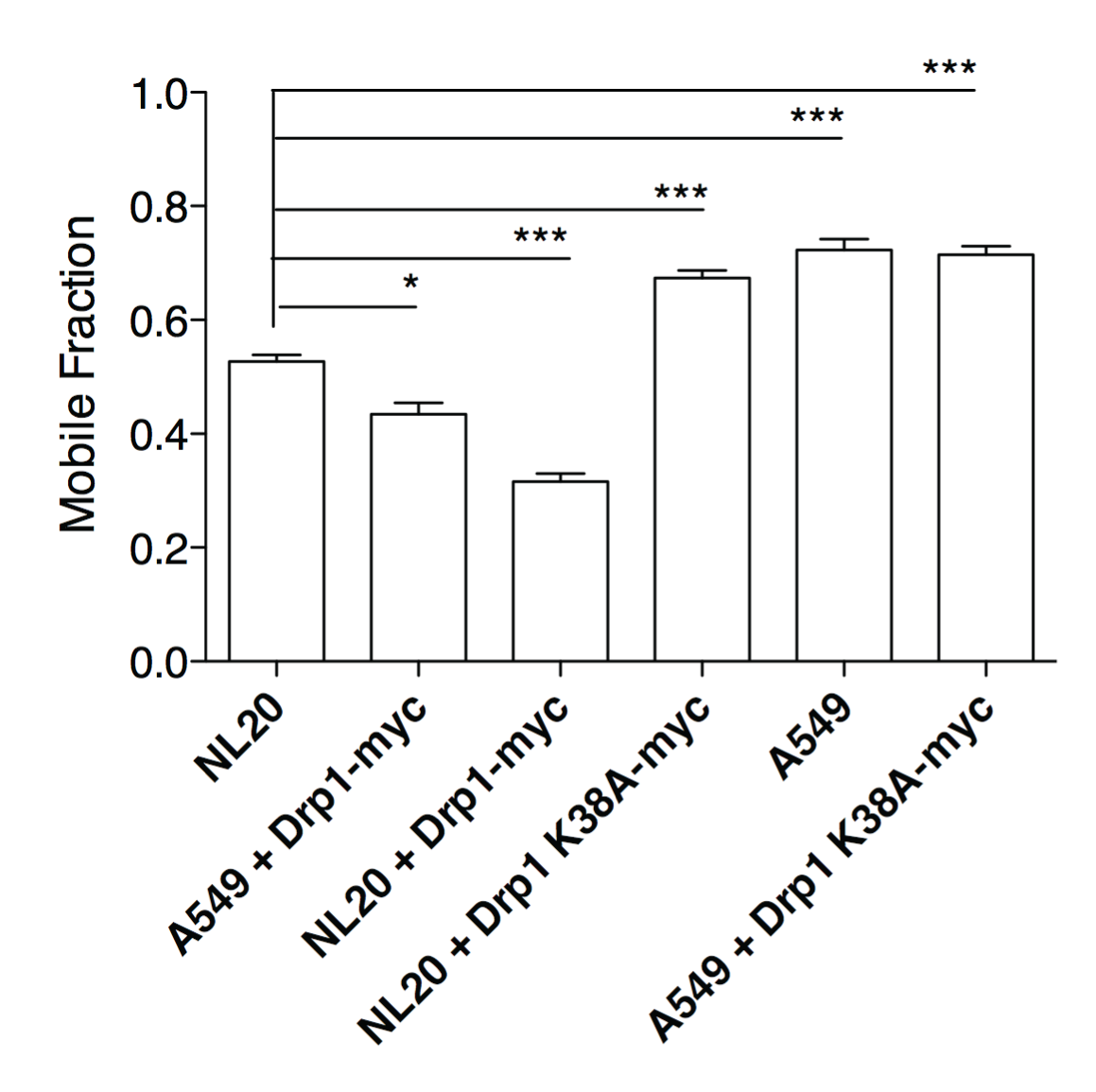

Supplement: Figure S2 — Additional FRAP analysis. Mobile fraction of mito-YFP values in NL20 and A549 cells are displayed which estimate mitochondrial connectivity or the relative amount of mitochondrial fission that is occurring in a single region of interest within the cell under basal, Drp1 K38A-myc downregulation or Drp1-myc overexpression. Mean and SEM shown from duplicate (n = 60 cell measurement) experiments. 1-way ANOVA analysis with Tukey post-tests. (TIF) [file pone.0045319.s002.tif]

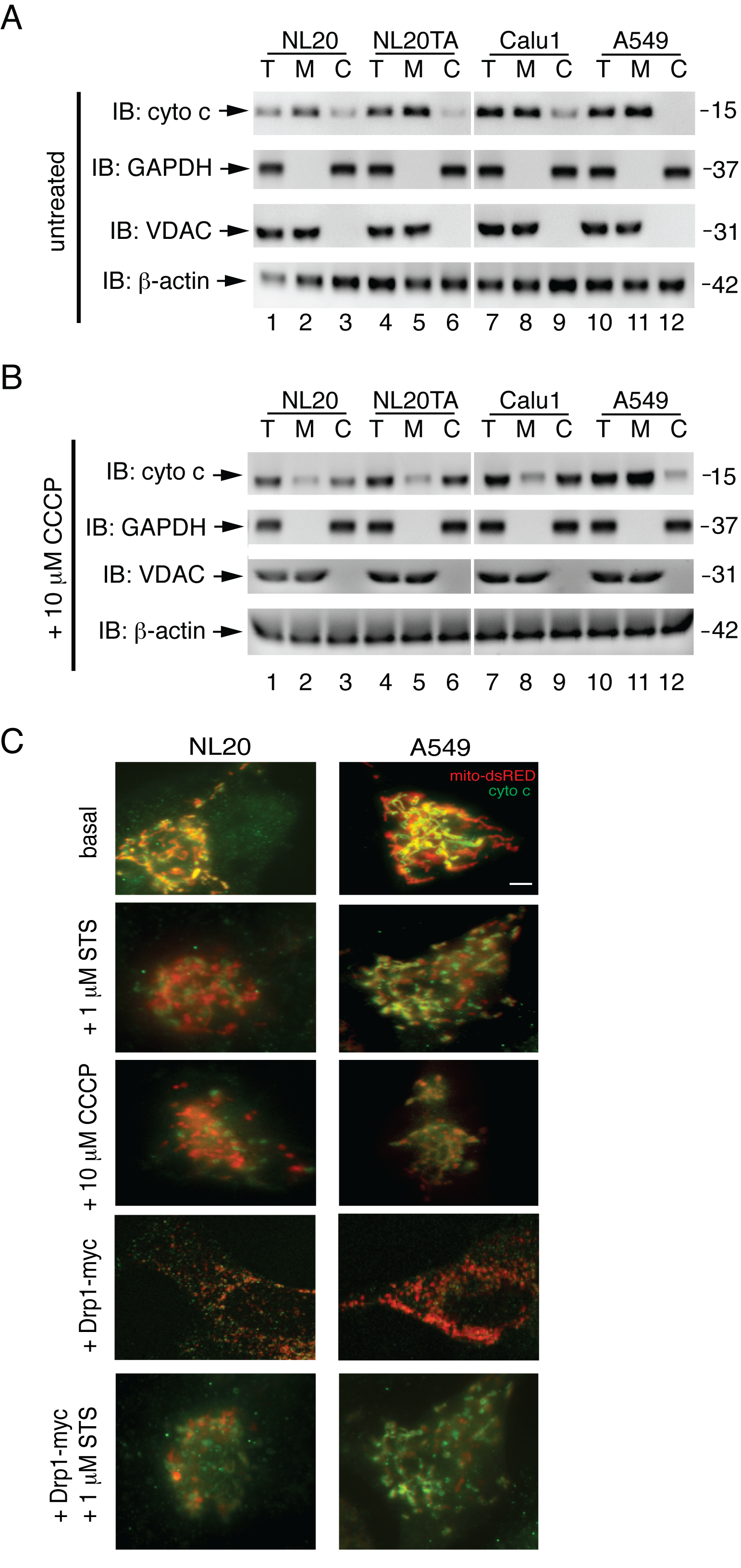

Supplement: Figure S3 — Cytochrome c release following mitochondrial uncoupling. (A,B) NL20 (lanes 1–3), NL20TA (lanes 4–6), Calu1 (lanes 7–9), and A549 (lanes 10–12) cells were harvested and subcellular fractionation was performed to examine mitochondrial and cytosolic fractions. Total (T: lanes 1,4,7,10), mitochondrial (M: lanes 2,5,8,11) and cytosolic (C: lanes 3,6,9,12) lysates were immunoblotted for endogenous cytochrome c. The mitochondrial (VDAC) and cytosol (GAPDH) markers and β-actin are shown as loading and fractionation controls. Markers are in kDa. (B) Cells were treated with 10 µM CCCP for 1 h to induce mitochondrial decoupling to examine cytochrome c release. (C) NL20 (left panels) and A549 (right panels) cells were transfected with mito-dsRED and immunostained for cytochrome c to show mitochondrial (red/orange) or cytoplasmic (green) localization. Colocalization of mitochondria and cytochrome c is indicated by yellow. Cells were untreated (top panels), treated with 1 µM STS for 3 h (second panels), treated with 10 µM CCCP for 1 h (third panels), co-transfected with Drp1-myc (fourth panels) or co-transfected with Drp1-myc and treated with 1 µM STS for 3 h (bottom panels). Scale bar is 2 µm. (TIF) [file pone.0045319.s003.tif]

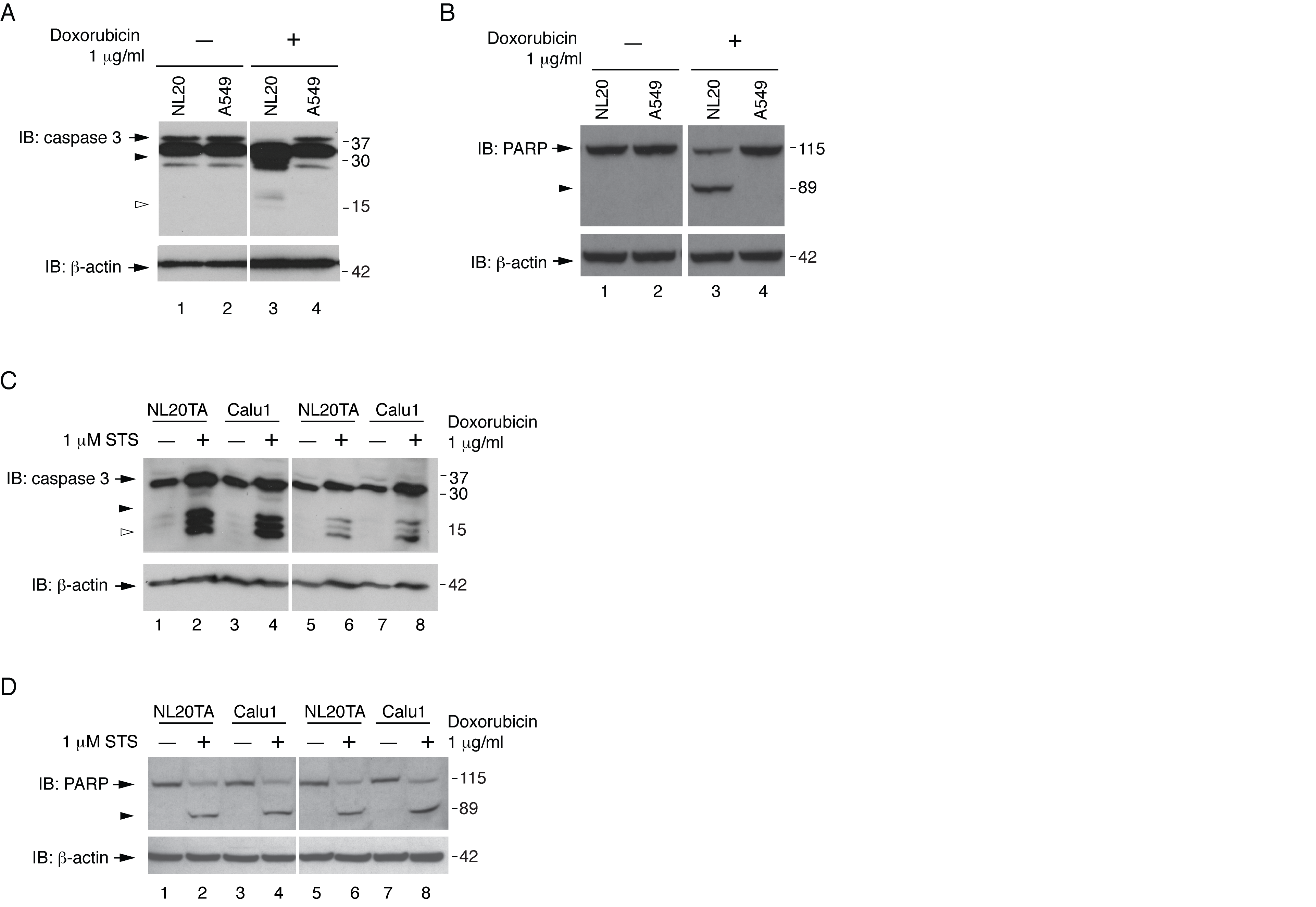

Supplement: Figure S4 — Apoptotic stimulus in epithelial cells. (A,B) NL20 (lanes 1,3) and A549 (lanes 2,4) cells untreated (lanes 1,2) or treated for 24 h with 1 µg/ml doxorubicin (lanes 3,4). β -actin reprobe to show loading. (C,D) NL20TA (lanes 1,2) and Calu1 (lanes 3,4) cells untreated (lanes 1,3) or treated for 3 h with 1 µM STS (lanes 2,4). NL20TA (lanes 5,6) and Calu1 (lanes 7,8) cells untreated (lanes 5,7) or treated for 24 h with 1 µg/ml doxorubicin (lanes 6,8). β -actin reprobe to show loading. (A,C) Immunoblots show endogenous caspase 3 protein expression (full length, arrow; cleaved products, open or closed arrowhead). (B,D) Immunoblots show endogenous PARP protein expression (full length, arrow; cleaved products, arrowhead). (A–D) Markers in kDa. (TIF) [file pone.0045319.s004.tif]

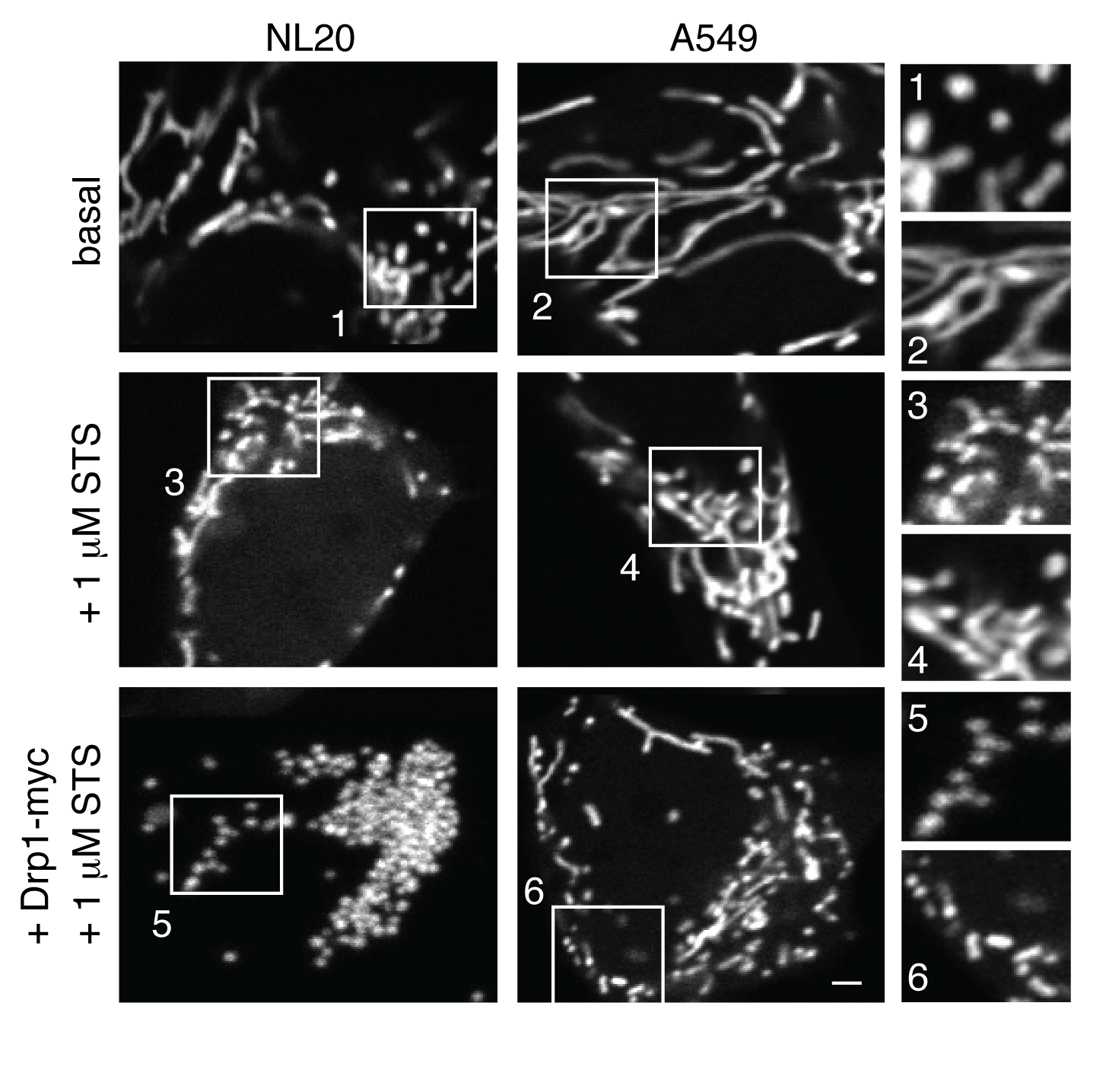

Supplement: Figure S5 — Mitochondrial morphology following STS exposure. (A) Mitochondrial morphology of NL20 (left panels) and A549 (right panels) cells following mito-YFP transfection. Representative images shown: basal (upper panels), and following 3 h treatment with 1 µM STS (middle panels) or Drp1-myc co-transfection with 3 h 1 µM STS treatment (lower panels). The scale bar indicates 2 µm. Box insets (numbered 1–6) to the far right are magnified (10×) regions corresponding to the numbered boxes in the original images. (TIF) [file pone.0045319.s005.tif]

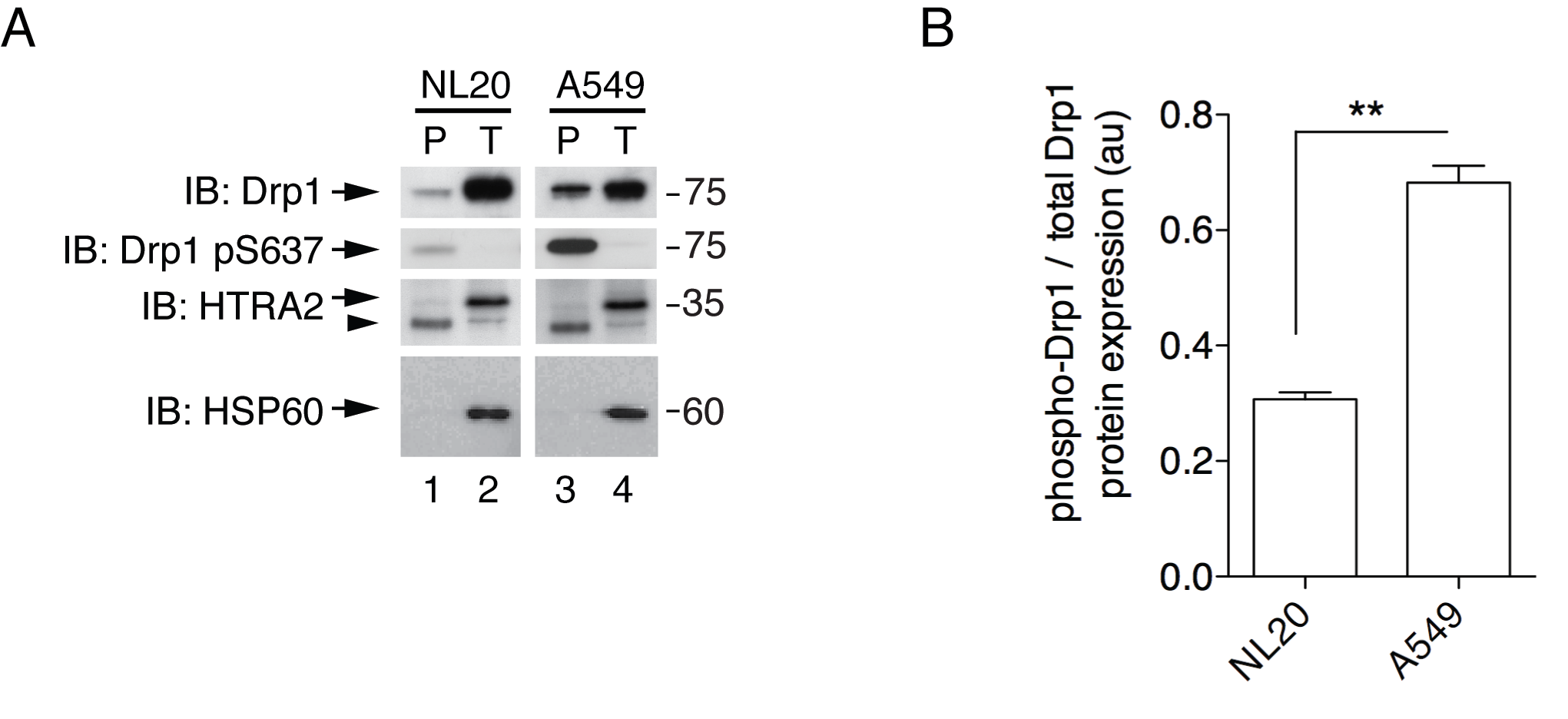

Supplement: Figure S6 — Analysis of Drp1 phosphorylation. (A) Cell lysates from NL20 and A549 cells were separated by phospho-enrichment (P; lanes 1,3) and compared to total lysates (T; lanes 2,4). With immunoblot, endogenous Drp1 was found in both phospho- and total fractions. Immunoblotting for Drp1 pSer637 was also included. Controls for purification include immunoblotting for non-phosphorylated HSP60 and phosphorylated HTRA2. Markers in kDa. (B) The ratio of phospho- to total Drp1 was quantified (B) from two independent experiments between the two cell lines. Mean and SEM shown. T-test analysis (P = 0.0071). (TIF) [file pone.0045319.s006.tif]
